# Fishing Technique of Long-Fingered Bats Was Developed from a Primary Reaction to Disappearing Target Stimuli

**DOI:** 10.1371/journal.pone.0167164

**Published:** 2016-12-14

**Authors:** Ostaizka Aizpurua, Antton Alberdi, Joxerra Aihartza, Inazio Garin

**Affiliations:** 1 Natural History Museum of Denmark, University of Copenhagen, Øster Voldgade, Copenhagen K, Denmark; 2 Department of Zoology and Animal Cell Biology, Faculty of Science and Technology, University of The Basque Country, UPV/EHU, Sarriena z/g, Leioa, The Basque Country, Spain; University of Western Ontario, CANADA

## Abstract

Behavioral plasticity is a key feature allowing animals to broaden their dietary niche when novel food resources become available, and long-fingered bats provide an appropriate model system to study the underpinnings of behavioral plasticity, since although generally being an insectivorous species, some individuals have been reported to catch fish. Aiming to get insight into the origin of fishing behavior in long-fingered bats, we studied in the field the differences in sensorial and mechanical reactions to insect-like (stationary) and fish-like (temporary) prey stimuli between well-known piscivorous and strictly insectivorous individuals. Both piscivorous and insectivorous individuals exhibited a qualitatively similar reaction to temporary target stimuli (longer and deeper dips and terminal echolocation phase skewed towards *buzz I* compared to stationary stimuli). Nevertheless, the quantitative differences observed in the sensorial and mechanical features (the intensity of the shift was significantly greater in piscivorous than in insectivorous individuals) show that piscivorous individuals have honed their capture technique likely enhancing the fishing success. Thus, our results suggest that the fishing technique was developed from a primary reaction shared by all long-fingered bats. All individuals seem to be mechanically and sensorially adapted to detect and capture fish, although under appropriate environmental conditions, they would further improve their technique by experience and/or social learning.

## Introduction

Environmental variation forces organisms to be in a continual process of adaptation. The ability to adjust to novel conditions faster or better than their competitors is what makes the difference in terms of individual survival [1]. In animals, behavior is at the forefront of such adaptive capacity. The ability of animals to modify their behavior, namely behavioral plasticity, is the result of the complex mixture of innate traits and those acquired through learning [2–5]. Animals seem to be born with a predisposition to perform a behavior, which is later modified to a larger or lesser degree by experience or learning [6]. For example, bats have an innate recognition of water [7] and an innate tendency to investigate an item [8], but self experience and social interaction with experienced bats plays an integral role in the development of foraging skills [8,9].

The European long-fingered bat (*Myotis capaccinii*) is especially interesting to study such processes. In spite of its widespread insectivorous nature, individuals with a behaviorally different foraging habit, namely fishing, have been reported in geographically isolated localities [10–12]. Piscivorous *M*. *capaccinii* have developed a specific feeding technique for capturing fish [13], and rely on the kinetic features of the prey to discern fish from insects [14]. Any body that remains stationary on the water-surface is attacked using a technique usually employed to hunt insects. In the final approach phase to the prey, bats modify their echolocation call through shortening of the pulse interval, widening of the bandwidth and lowering of call-end-frequency. Two parts of this terminal phase can be distinguished, namely *buzz I* and *buzz II*. When catching insects from the water surface both parts of the terminal phase have similar length and the bats perform superficial and short dips. Conversely, temporary targets that disappear under the water during the capture act are attacked with deep and long dips, and a *buzz I*-biased terminal phase, which is a technique commonly used to catch fish [13]. Although the main ecological and sensorial characteristics of fishing have been addressed, it is not yet clear whether recognition of fish and the subsequent capture technique is a primary ability shared by all *M*. *capaccinii*, or the result of local learning processes limited to a few colonies. If recognition and capture of fish were a primary ability of all *M*. *capaccinii* we would not expect response differences between piscivorous and insectivorous individuals, because all bats would be able to recognize the disappearing stimulus as fish; thus, all of them would employ the fishing technique. In contrast, if fishing ability were acquired by experience and/or learning only by fish-eating individuals, insectivorous individuals would not recognize the disappearing stimulus as fish and their response would be similar to that exhibited when capturing insects.

In this study, we compare the sensorial and motor responses of fishing and non-fishing individuals (hereafter “piscivorous” and “insectivorous” individuals respectively) to different stimuli, to address whether the recognition and capture of fish is a primary ability shared by all long-fingered bats, or a specific ability just developed by piscivorous individuals.

## Materials and Methods

### Study area

The field study was carried out in June-July 2012 at two different locations in Western Iberian Peninsula: a pond in “La Sella” golf course (Dénia, Alacant) and a stream pool at Vernissa River (Ròtova, Valencia). Both sites are large (> 10 m width) water fields without ripples or aquatic vegetation on the surface, and common foraging grounds used by *M*. *capaccinii* from different colonies. They are located 32 km apart from each other, beyond the maximum foraging range recorded for the species [15] and a radio-tracking study [16] as well as the lack of captures in each cave of individuals ringed in the other colony (unpublished data) suggest small or nonexistent contact between both populations. The first pond, full of *Gambusia holbrooki* fish and located in a golf course, is the only site reported as fishing ground of the long-fingered bat in the Iberian Peninsula [17]. The second experimental site is a foraging spot commonly used by *M*. *capaccinii* roosting in another cave where no traces of piscivory have been found so far. There are not *Gambusia* or other similar surface-feeder fish in this second site. Therefore, the animals foraging in “La Sella” golf course are named as “piscivorous individuals”, while bats feeding in Vernissa River are named as “insectivorous individuals”.

### Bulk guano analysis

To ensure allegedly insectivorous individuals have not eaten fish, we looked for fish traces (scales and otoliths) in *ca*. 5 kg of guano obtained three months prior the study under the long-fingered bats aggregation using a method based on physical filtering. We left the sample to soak for 24 hours in water to homogenize the sample and filtered using three sieves of different size (2 mm, 1 mm and 0.5 mm), from coarse to fine. Efficiency of the method was previously verified by mixing 6 otoliths and scales of the potential prey fish *G*. *holbrooki* (the species *M*. *capaccinii* consumes in the wild [17]) in a 2 kg bulk of *Myotis myotis* guano (strictly insectivorous species), from which we recovered 5 otoliths and detected the presence of scales.

### Experimental setup and recording analysis

We performed two experiments during 10 nights in each of the experimental sites, i.e. in the foraging ground of piscivorous individuals and in the foraging ground of insectivorous individuals. In the first experiment, we presented on the water surface two different stimuli: a stationary and a temporary fish. To represent the stationary target we tethered a dead eastern mosquitofish *G*. *holbrooki* from the abdomen with small tweezers. To represent the temporary target we also used a dead eastern mosquitofish, but tethered to a custom mechanism that would cause continually the target to either lightly protrude from the water’s surface for one second or submerse it for two seconds. The instrument submerged the fish down totally, bringing the fish out of reach or any contact of the bat’s feet (supporting information S1 Fig). Only the attacks performed when the target was submersed were considered as attacks upon temporary targets. Thus, bats did not get any somatosensory feedback from the prey when attacking upon temporary targets. In the second experiment, instead of submerging the prey every two seconds, it was submersed manually at different moments along the bat’s target pursuit trajectory (20–300 ms before prey contact) in order to analyze how piscivorous and insectivorous *M*. *capaccinii* respond to target disappearance and to find out their reaction ability. A normal-speed digital camcorder (Sony HDR550, Sony Corporation, Tokyo, Japan) in nightshot mode was used to control the approaching of the bats and decide when to submerge the target. In all cases fish were tethered (impossible to be caught by bats) and placed under water with their upper lip breaking the surface, mimicking the natural surface-feeding behavior of eastern mosquitofish. Fish were caught with a hand-net in the artificial pond in “La Sella” golf course and killed by cervical dislocation before performing the experiment [18]. We recorded hunting attempts using a high-speed (500 frames per second) video camera (HiSpec, Fastec imaging Corporation, USA), infrared light torches (IREL-45, ECV Video Seguridad S.A., Sabadell, Catalonia) and an ultrasound detector (D1000X, Pettersson Elektronik AB, Uppsala, Sweden). The sound and video inputs were synchronized using an electronic clapper (further details in [13]). In order to prevent potential pseudo-replication, we only performed the experiments in the presence of >10 long-fingered bats in the experimental ground, and we used a wide-angle digital camcorder to track the flight of the bats and ensure that consecutive capture attempts were performed by at least 5 different individuals.

Sound analyses were performed using BatSound (Petterson Elektronik AB, Uppsala, Sweden) and only the terminal phase of the echolocation sequence (continuous sequence of calls emitted by the bat just before a capture attempt) was analyzed. The terminal phase is produced after a pre-buzz pause [13] and is divided into two parts: *buzz I* and *buzz II*. *Buzz II* can be differentiated from *buzz I* due to its distinct drop in the peak frequency. We measured the total pulse number of the terminal phase, as well as the pulse number of *buzz I* and *II*. Video recordings were analyzed using Fastec software (Fastec imaging Corporation, USA). For each recording we measured the total dip duration (lapse in which the feet is in contact with the water), the feet insertion depth (the extent to which the hind feet are inserted into the water, see supporting information S2 Fig) and the binary response of whether bats move or not the head toward the tail membrane/feet after the capture attempt (feet-mouth movement).

### Attack characteristics

We first compared the six variables—(1) the total pulse number, (2) the number of *buzz I* pulses, (3) the number of *buzz II* pulses, (4) the duration of the dip, (5) the depth of the dip and (6) the feet-mouth movement—of the attacking action in piscivorous and insectivorous long-fingered bats. Variables confirmed as normally distributed by a Kolmogorov-Smirnov test were analyzed using one-way Student t-tests (t-test) and Mann-Whitney test (M-W) was used for variables not fulfilling the assumption of normality. Pearson’s Chi-Squared test (χ^2^) was used to compare frequencies of dip depth and feet-mouth movement variables. Subsequently, we ran a principal component analysis (PCA) to identify the most meaningful axes and visualize the response of bats to different stimuli in one and two dimension charts. All the statistical analyses were performed in R 3.0.2 (http://cran.r-project.org/).

### Response to target disappearance

The differences in response between piscivorous and insectivorous individuals to target disappearance were analyzed using reaction norms. Reaction norms are functions describing the change in the phenotype (in our case behavior) across an environmental gradient, and are broadly employed to model phenotypic plasticity [19,20]. In our experimental approach, the fish disappearance moment played the role of environmental gradient, and we observed the variation of the above-mentioned variables when the target was removed at different instances during the attack sequence. We generated reaction norms using linear regression, where the elevation (the intercept in statistical terms) of the regression line represents the response value exhibited in the average environment, while the slope exhibits the behavioral plasticity. Slopes of the regression of different piscivorous and insectivorous individuals were compared using analysis of covariance (ANCOVA) in GraphPad Prism (GraphPad Software, San Diego, USA).

### Ethics statements

Fish capture and handling protocols met the guidelines for treatment of animals in research and teaching [18]. The study met Spanish legal requirements and was approved by the Ethics Committee for Animal Welfare of the University of the Basque Country (Refs. CEBA/220/2012/AIHARTZA and CEBA/221/2012/AIHARTZA).

## Results

### Attack characteristics

**Fig 1:**
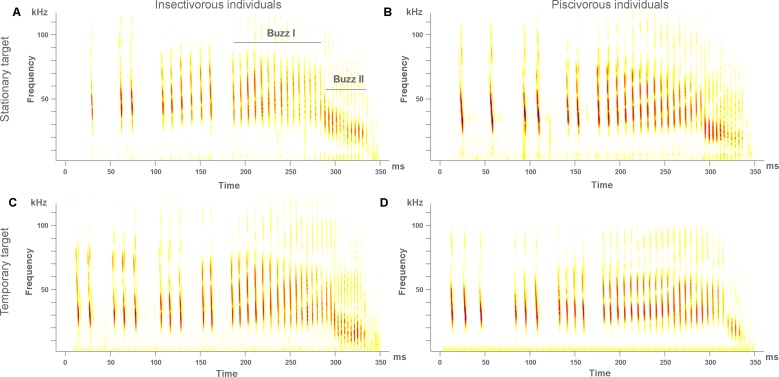
Illustrative spectrograms of the terminal echolocation phase. (A-B) Insectivorous and piscivorous individuals exhibit almost identical echolocation patterns when attacking stationary targets. In both cases the number of *buzz I* pulses is slightly higher than the number of *buzz II* pulses. (C) Insectivorous individuals vary their echolocation pattern minimally when attacking temporary targets, maintaining the number of *buzz I* pulses, and slightly decreasing the number of *buzz II* pulses. (D) In contrast, piscivorous individuals noticeably decrease the number of *buzz II* pulses while increasing the number of *buzz I* pulses when attacking temporary targets with respect to attacks upon stationary targets.

**Fig 2:**
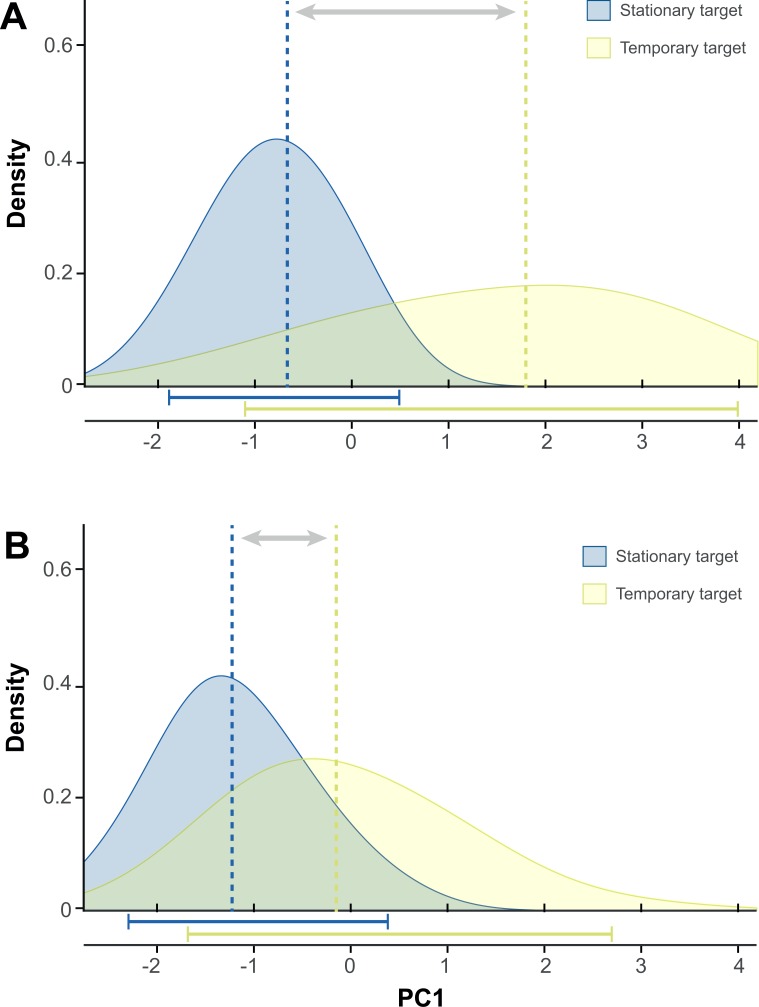
**Displacement of the density curves of the Principal Component 1 (PC1) between attacks on stationary (blue) and temporary (yellow) targets by (A) piscivorous individuals and (B) insectivorous individuals.** Vertical dashed lines indicate median values, and horizontal solid lines indicate area within the 5% and 95% percentiles. Note that the displacement is larger in the case of piscivorous bats (A). A two-dimensional representation of the PCA is shown in supporting information S3 Fig.

In the first experiment we recorded 298 synchronized high-quality echolocation audios and high-speed videos of capture attempts upon stationary and temporary targets, 143 of piscivorous individuals and 170 of insectivorous individuals. We observed that both piscivorous and insectivorous individuals performed similar attacks upon stationary targets, and both varied their technique when attacking temporary targets. The detailed analysis of each variable showed that in both piscivorous and insectivorous individuals dip depth (supporting information S2 Fig) and dip duration increased from stationary to temporary target attempts, while the number of *buzz II* pulses decreased (Table 1, Fig 1). However, a considerably larger displacement of the attack characteristics from stationary to temporary target can be observed in piscivorous individuals than in insectivorous individuals (Fig 2, supporting information S3 Fig), due to two main reasons. First, the number of *buzz I* pulses and the feet-mouth movement differed between stationary and temporary target attempts in piscivorous individuals, yet remained equal in insectivorous individuals; and second, the displacement of dip depth and *buzz II* pulse number was stronger in the piscivorous individuals (Fig 1). Dip duration was the only variable that showed the opposite pattern, i.e. larger difference in insectivorous individuals (Table 1).

**Table 1:** General features of the terminal phase and dip pattern measured during piscivorous and insectivorous individuals attacks on the stationary and temporary targets. (A) Differences in the stationary target attack features between piscivorous and insectivorous individuals (*N* = 151), (B) differences in the temporary target attack features between piscivorous and insectivorous individuals (*N* = 154) and (C) statistical tests between stationary and temporary target attack features in each group. *Entire feet insertion* refers to the relative proportion of attacks where the entire feet were inserted into the water. The detailed measurements of dip depth are shown in the supporting information S2 Fig. *Feet-mouth movement* is a binary measure of whether bats move or not the head toward the tail membrane/feet after the capture attempt.

**A**			
**Attack on stationary target**	**Insectivorous**	**Piscivorous**	**Statistics**
	***N* = 77**	***N* = 74**	
**Total number of pulses**	26 ± 3.60	26 ± 4.01	t-test: *t* = -0.11, *P* = 0.916
**Number of pulses in *buzz I***	15 ± 3.28	15 ± 3.55	t-test: *t* = -0.87, *P* = 0.931
**Number of pulses in *buzz II***	11 ± 1.74	11 ± 1.90	M-W: *U* = 2858, *P* = 0.854
Dip duration (ms)*	15.8 ± 13.41	25.5 ± 10.33	M-W: *U* = -3.542, P < 0.010
Entire feet insertion (%)*	0	9	χ^2^(1) = 23.61, *P* < 0.010
Feet-mouth movement (%)*	73	89	χ^2^(1) = 6.40, *P* < 0.010
**Results are presented as mean ± standard deviation and asterisks indicate significant differences between the two groups (P < 0.05).**			
**B**			
**Attack on temporary target**	**Insectivorous**	**Piscivorous**	**Statistics**
	***N* = 85**	***N* = 69**	
**Total number of pulses**	25 ± 4.89	25 ± 3.53	t-test: *t* = -0.74, *P* = 0.459
Number of pulses in buzz I*	15 ± 3.92	17 ± 3.84	t-test: *t* = -2.29, *P* = 0.023
Number of pulses in buzz II*	10 ± 2.67	7 ± 3.71	t-test: *t* = 3.47, *P* < 0.001
Dip duration (ms)*	34.2 ± 15.98	42.6 ± 15.65	t-test: *t* = 2.79, *P* < 0.010
Entire feet insertion (%)*	3	56	χ^2^(1) = -0.46, *P* < 0.001
Feet-mouth movement (%)*	74	44	χ^2^(1) = -0.21, *P* < 0.010
**Results are presented as mean ± standard deviation and asterisks indicate significant differences between the two groups (P < 0.05).**			
**C**			
**Stationary vs. Temporary**	**Insectivorous**		**Piscivorous**
	***N* = 172**		***N* = 143**
**Total number of pulses**	t-test: *t* = 1.58, *P* = 0.125		t-test: *t* = 1.6, *P* = 0.106
**Number of pulses in *buzz I***	t-test: *t* = -0.61, *P* = 0.541		t-test: *t* = -3.2, *P* = 0.001*
**Number of pulses in *buzz II***	t-test: *t* = 3.79, *P* < 0.001*		M-W: *U* = 878.5, *P* < 0.001*
**Dip duration (ms)**	M-W: *U* = 5503, *P* < 0.001*		t-test: *t* = 6.6, *P* < 0.001*
**Entire feet insertion (%)**	χ^2^(1) = 50.15, *P* < 0.001*		χ^2^(1) = 36.6, *P* < 0.001*
**Feet-mouth movement (%)**	χ^2^(1) = 0.06, *P* = 0.807		χ^2^(1) = 33.4, *P* < 0.001*
**Asterisks indicate significant differences between stationary and temporary targets (P < 0.05).**			

### Response to target disappearance

**Fig 3:**
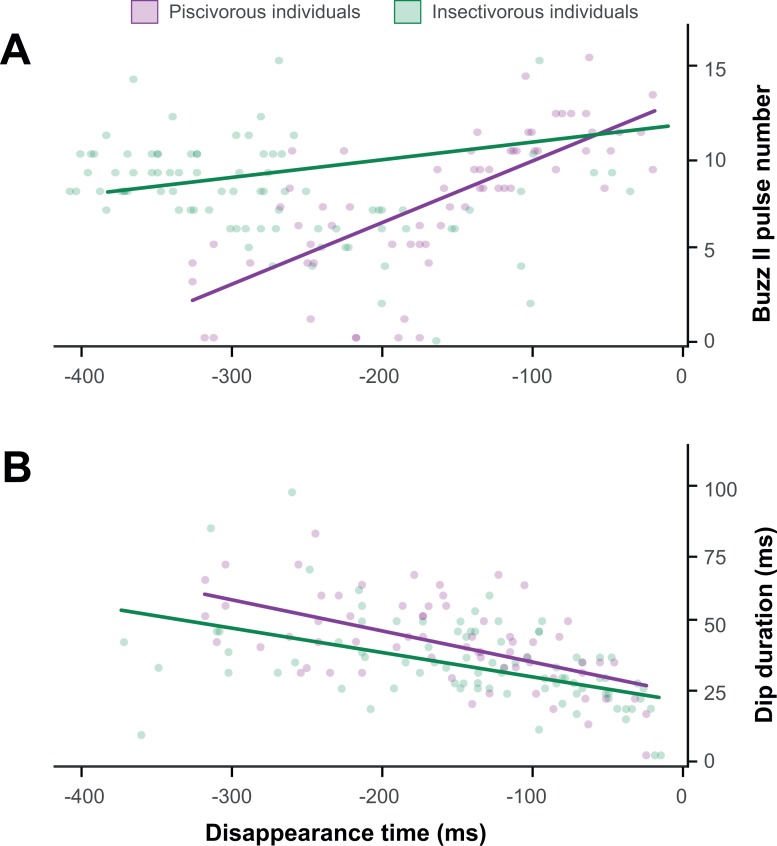
**The relationships between (A) number of pulses in *buzz II* and fish disappearance time and (B) dip duration and fish disappearance time.** The moment of capture is presented as 0 ms (on the right side of the graph). (A) The sooner disappearance of the target produces a decrease in the number of *buzz II* pulses in both piscivorous and insectivorous individuals, even though the trend is more pronounced in piscivorous individuals. (B) The sooner disappearance of the target produces a similar increase in the duration of the dip in both piscivorous and insectivorous individuals.

The target disappearance time was only correlated with *buzz II* pulse number and dip duration, and the analyses of 162 recording attempts (piscivorous = 69, insectivorous = 93) showed that the earlier the disappearance of the fish, the longer the dip duration and the less the number of *buzz II* pulses in both piscivorous and insectivorous individuals. However, while dip duration variation was similar in both cases (ANCOVA: *F*_1,149_ = 0.22, *P* = 0.640), the effect of fish removal on the amount of pulses of *buzz II* was stronger in the piscivorous individuals (ANCOVA: *F*_1,146_ = 24.99, *P* < 0.001) (Fig 3).

## Discussion

We analyzed the fishing skills of long-fingered bats by comparing piscivorous individuals to insectivorous individuals, to better understand the underpinnings of fishing ability. When we conceived the study, we assumed that similar responses between piscivorous and insectivorous individuals to fish-like stimuli would indicate a primary ability to recognize and capture fish shared by all long-fingered bats, while insectivorous individuals responding to fish-like stimuli using insect-capture-like attacks would indicate that experience and/or learning played an important role in piscivorous individuals in modifying the hunting technique to catch fish. However, the data do not support any of these utmost assumptions, but suggest an intermediate scenario. Both piscivorous and insectivorous individuals use different techniques to capture insects and to catch fish, but the fishing technique of piscivorous individuals also differs from that of insectivorous individuals. Hence, there is a shared primary ability to react to a disappearing target, but unlike insectivorous individuals, piscivorous individuals have honed their attack technique for fishing.

Both piscivorous and insectivorous long-fingered bats modify their attack pattern when the target disappears under the water. They make deeper and longer dips when the prey disappears during the attack sequence. Although we cannot confirm that insectivorous individuals recognize the temporary target as fish, the tendency to make deeper and longer dips when the prey suddenly disappears seems to suggest part of the natural repertoire of all long-fingered bats, regardless of their dietary habits. Bats also make a slight modification in their echolocation calls, decreasing the number of *buzz II* pulses when the target disappears. A recent study reported similar reactions in another trawling bat, *M*. *daubentonii*, suggesting that our observations in *M*. *capaccinii* might extend to other trawling bats too [21].

All bats in the study modify their attack pattern when facing temporary targets, but the contrast between attacks upon stationary and temporary targets is stronger in piscivorous than in insectivorous individuals. This pattern is observed in all analyzed variables except dip duration. Although piscivorous individuals perform considerably longer dips when attacking temporary targets, the difference between attacks upon stationary and temporary targets is greater in insectivorous individuals. This is produced by the much lower average dip duration of insectivorous individuals when attacking stationary targets (15.8 ms vs. 25.5 ms in piscivorous individuals). This lower value is the result of the high number of attacks on the prey without touching the water (thus dip duration = 0) in insectivorous individuals. The sensorial features of insectivorous individuals also showed very little variation between stationary and temporary targets, while the difference was considerable in piscivorous individuals. Piscivorous individuals decreased the number of *buzz II* pulses in a more pronounced manner than insectivorous individuals when the target disappeared, exhibiting steeper reaction norms that indicate increased plasticity [20]. Additionally, unlike the insectivorous, the piscivorous individuals increased the number of *buzz I* pulses, maintaining the total feeding buzz pulse number constant. This fact suggests that fishing long-fingered bats would have learned that *buzz I* extension provides advantages. The higher frequency and larger bandwidth of *buzz I* calls generate a narrower sonar beam and a higher echo strength [14,22,23]. In the last instances of the capture process, these features might provide better tracking ability of the exact location where the fish disappeared and a clearer perception of the ripples produced by the submerged fish, thus improving fish location capacity and increasing fishing efficiency.

Interestingly, the greatest difference between piscivorous and insectivorous individuals was observed in the post-capture behavior when the bats moved their feet to the mouth. Although the target was stuck and thus could not actually be *hunted* but touched, both piscivorous and insectivorous individuals showed such behavior when attacking stationary targets, suggesting that the movement is an automatic response when hunting stationary targets like insects laid on the water surface to transfer prey to the mouth. However, while piscivorous individuals did not display the same movement when attacking temporary targets, insectivorous individuals kept exhibiting the same behavior. When attacking temporary targets bats did not touch the prey, so in our opinion this behavior indicates that piscivorous individuals have learned to respond to a different somatosensory feedback. These bats seem to be aware that they are about to capture a heavy item (fish are up to 50-fold heavier than insects) and therefore do not move their feet to their mouth when they do know that the capture attempt has failed. In contrast, insectivorous bats keep exhibiting the same behavior of moving their feet to their mouth as when attacking stationary targets, suggesting that they are not aware of what they are hunting.

The observed variations suggest that insectivorous individuals instinctively modify their hunting pattern when the target disappears during the attack, but unlike the piscivorous individuals, they have not fully developed the technique to capture fish. Thus, fishing bats have honed their fishing technique from a primary reaction to disappearing target stimuli. Consequently, long-fingered bats do have preadaptations that can make fishing possible if the necessary conditions are met, but they have the ability—and probably the need, to hone their attack features to make fishing cost-effective. Although our study does not provide clues about the time needed to learn specialized fishing technique, the only ponds—according to a radio-tracking study [17]—the piscivorous individuals use for fishing were built between 2002 and 2009. The first evidence of fish consumption was reported in 2003 [12], and the first video recordings where bats could be observed fishing were taken in 2009 [17]. This scenario would suggest that as soon as high density of surface-feeder fish become available bats are able to exploit them. This possibility is supported by observations carried out in captivity [24], where insectivorous *M*. *capaccinii* bats, after a short period of acclimation, were able to capture fish from an artificial pond with large amounts of superficial fish. However, whether such improvements in the hunting technique to enable the effective capture of fish can happen in a very short time or need several generations of social learning is an interesting future research avenue that may cast light on learning processes in mammals.

## Supporting Information

S1 Fig **Schematic illustration of the operation of the (A) stationary and (B) temporary targets.** (B.1) When the fishing line was pulled the fish was submerged, and (B.2) when the fishing line was released the buoyancy of the cork caused the emergence of the upper lip of the fish.
(PDF)

S2 Fig Differences in the feet insertion depth between different bats (insectivorous vs. piscivorous) and target types (stationary vs. temporary). This feature was classified into three categories: touching the water with the toes (toes), insertion of half of the foot into the water (half foot) and submersion of more than half of the foot into the water (entire foot).
(PDF)

S3 Fig **The principal component analysis (PCA) between attacks on stationary (blue) and temporary (yellow) targets by (A) piscivorous individuals and (B) insectivorous individuals.** The ellipses are drawn at a confidence level of 0.95. PC1 explains 47% of the variation, and the PC2 17%, for a cumulative proportion of 65%.
(PDF)
