# Supplementary material for: Fishing Technique of Long-Fingered Bats Was Developed from a Primary Reaction to Disappearing Target Stimuli
Source: PLoS One. 2016 Dec 14;11(12):e0167164. doi: 10.1371/journal.pone.0167164 (PMC5156352; doi:10.1371/journal.pone.0167164)

S1 Fig. Schematic illustration of the operation of the stationary (A) and temporary (B) target. (B.1) When the fishing line was pulled the fish was submerged, and (B.2) when the fishing line was released the buoyancy of the cork caused the emergence of the upper lip of the fish.

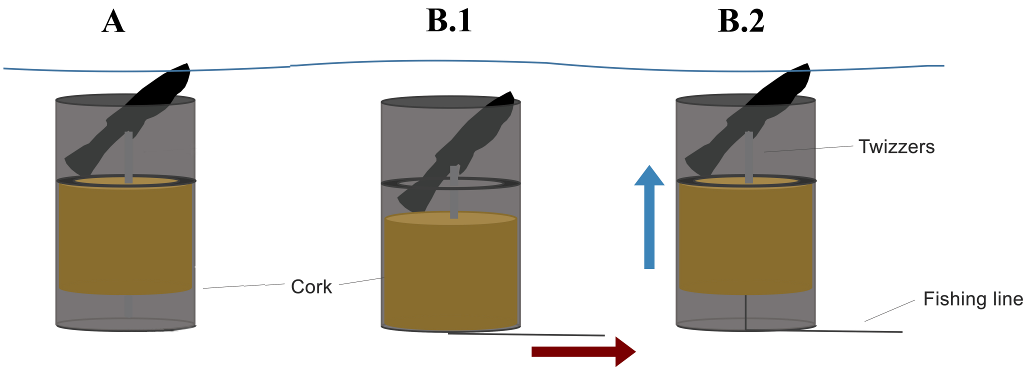

Supplement: S1 Fig — Schematic illustration of the operation of the (A) stationary and (B) temporary targets. (B.1) When the fishing line was pulled the fish was submerged, and (B.2) when the fishing line was released the buoyancy of the cork caused the emergence of the upper lip of the fish. (PDF) [file pone.0167164.s001.pdf]
