# Supplementary material for: Fishing Technique of Long-Fingered Bats Was Developed from a Primary Reaction to Disappearing Target Stimuli
Source: PLoS One. 2016 Dec 14;11(12):e0167164. doi: 10.1371/journal.pone.0167164 (PMC5156352; doi:10.1371/journal.pone.0167164)

S2 Fig. Differences in the feet insertion depth between different bats (insectivorous vs. piscivorous) and target types (stationary vs. temporary). This feature was classified into three categories: touching the water with the toes (toes), insertion of half of the foot into the water (half foot) and submersion of more than half of the foot into the water (entire foot)

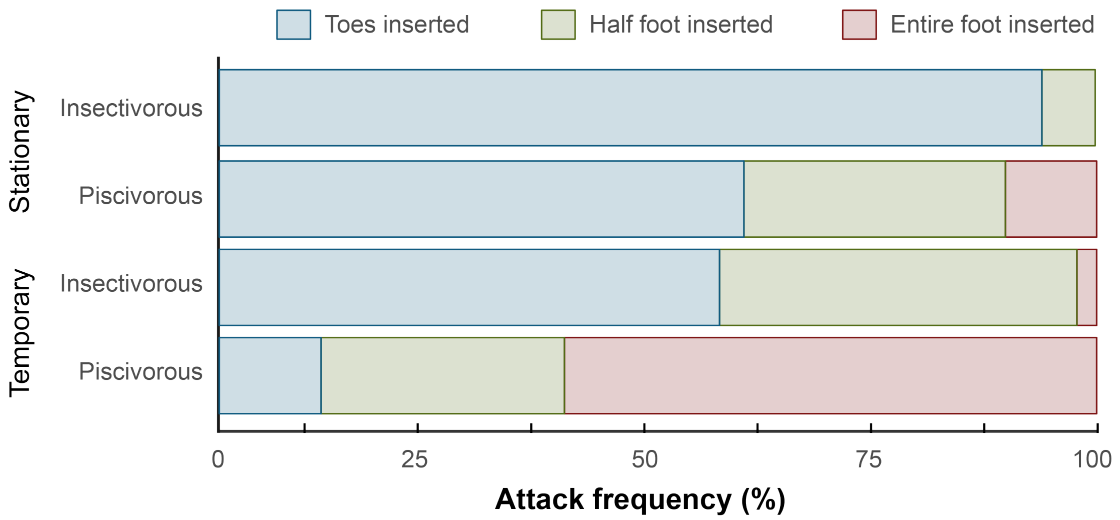

Supplement: S2 Fig — This feature was classified into three categories: touching the water with the toes (toes), insertion of half of the foot into the water (half foot) and submersion of more than half of the foot into the water (entire foot). (PDF) [file pone.0167164.s002.pdf]
