# Supplementary material for: Fishing Technique of Long-Fingered Bats Was Developed from a Primary Reaction to Disappearing Target Stimuli
Source: PLoS One. 2016 Dec 14;11(12):e0167164. doi: 10.1371/journal.pone.0167164 (PMC5156352; doi:10.1371/journal.pone.0167164)

S3 Fig. The principal component analysis (PCA) between attacks on stationary (blue) and temporary (yellow) targets by (A) piscivorous individuals and (B) insectivorous individuals. The ellipses are drawn at a confidence level of 0.95. PC1 explains 47% of the variation, and the PC2 17%, for a cumulative proportion of 65%

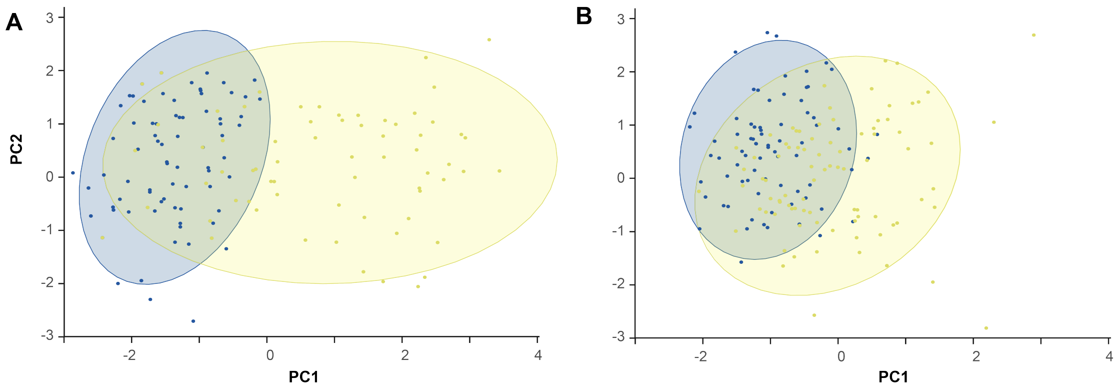

Supplement: S3 Fig — The principal component analysis (PCA) between attacks on stationary (blue) and temporary (yellow) targets by (A) piscivorous individuals and (B) insectivorous individuals. The ellipses are drawn at a confidence level of 0.95. PC1 explains 47% of the variation, and the PC2 17%, for a cumulative proportion of 65%. (PDF) [file pone.0167164.s003.pdf]
